# Supplementary material for: The interactome of intact mitochondria by cross-linking mass spectrometry provides evidence for coexisting respiratory supercomplexes
Source: Mol Cell Proteomics. 2017 Dec 8;17(2):216–32. doi: 10.1074/mcp.RA117.000470 (PMC5795388; doi:10.1074/mcp.RA117.000470)
Supplement: Supplemental Data [file supp_RA117.000470_133922_0_supp_23978_fzffwf.zip › spectra_annotation/mito_spectra_annotation/452-1-10-1-14-1.pdf]

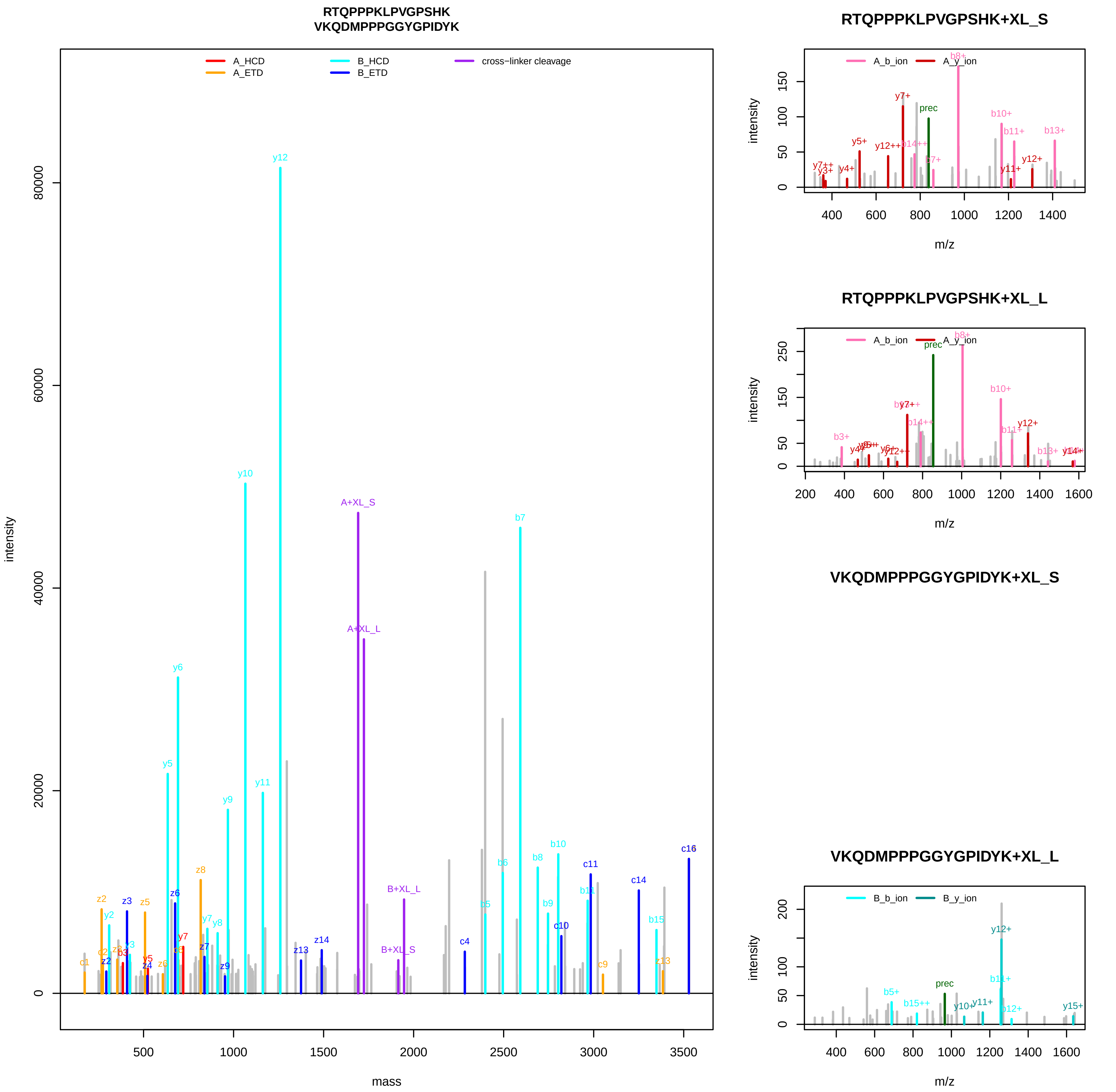

RTQPPPKLPVGPSHK
VKQDMPPPGGYGPIDYK
A_HCD
A_ETD
B_HCD
B_ETD
cross−linker cleavage
intensity
mass
y12
y10
A+XL_S
A+XL_L
b7
y6
y5
y11
y9
B+XL_L
B+XL_S
c16
c11
c14
b10
b8
b6
b5
b9
c10
b11
b15
z13
c9
c4
z14
z13
z9
z7
z8
y7
y8
z6
z5
z3
z2
y2
y3
c1
RTQPPPKLPVGPSHK+XL_S
RTQPPPKLPVGPSHK+XL_L
VKQDMPPPGGYGPIDYK+XL_S
VKQDMPPPGGYGPIDYK+XL_L
A_b_ion
A_y_ion
B_b_ion
B_y_ion
prec
m/z
